# Modelling bias in combining small area prevalence estimates from multiple surveys

**DOI:** 10.1111/j.1467-985X.2010.00648.x

**Published:** 2011-01

**Authors:** Giancarlo Manzi, David J Spiegelhalter, Rebecca M Turner, Julian Flowers, Simon G Thompson

**Affiliations:** Medical Research Council Biostatistics UnitCambridge, UK, and Università degli Studi di Milano, Italy; Medical Research Council Biostatistics UnitCambridge, UK; Eastern Region Public Health ObservatoryCambridge, UK; Medical Research Council Biostatistics UnitCambridge, UK

**Keywords:** Bias modelling, Hierarchical models, Meta-analysis, Mixed effects models, Multiple survey data, Small area estimation, Smoking prevalence

## Abstract

**Summary:**

Combining information from multiple surveys can improve the quality of small area estimates. Customary approaches, such as the multiple-frame and statistical matching methods, require individual level data, whereas in practice often only multiple aggregate estimates are available. Commercial surveys usually produce such estimates without clear description of the methodology that is used. In this context, bias modelling is crucial, and we propose a series of Bayesian hierarchical models which allow for additive biases. Some of these models can also be fitted in a classical context, by using a mixed effects framework. We apply these methods to obtain estimates of smoking prevalence in local authorities across the east of England from seven surveys. All the surveys provide smoking prevalence estimates and confidence intervals at the local authority level, but they vary by time, sample size and transparency of methodology. Our models adjust for the biases in commercial surveys but incorporate information from all the sources to provide more accurate and precise estimates.

## 1. Introduction

Small area estimates of the prevalence of risk factors are important for decision and policy makers, and their quality is a crucial concern. One example is in health promotion, when addressing area-specific health issues or lifestyle behaviours. In some deprived areas people might have more restricted access to screening programmes or preventive healthcare campaigns, or they may have a higher level of certain risk factors. Knowledge of the prevalence of risk factors in small areas is essential to make health promotion strategies more effective.

Classical solutions for small area estimation from multiple surveys (e.g. Lohr and Rao (2006), Moriarity and Scheuren (2001) and Elliott and Davis (2005)) have a practical drawback that they require access to individual level data. The day-to-day work of research agencies, however, relies on aggregate estimates from different sources. The customary approach to such problems is to consider only surveys that are carried out by official statistical agencies. Commercial surveys are rarely taken into account, because their sampling scheme or method of interviewing may cause bias and the detail of the methodology is often unclear or unavailable. However, they are updated more frequently than official surveys and can provide information on trends over time and estimates at a more local level.

In this context, modelling the bias in the various data sources is crucial and Bayesian analysis provides a convenient framework to incorporate all the available evidence (Turner *et al.*, 2009; Spiegelhalter and Best, 2003). We propose a series of models to address this problem, which allow for additive biases both within and between sources of data. A classical counterpart of these models is also presented, by using a mixed effects analysis approach.

We apply these models to obtain bias-adjusted estimates of smoking prevalence in local authorities (LAs) across the east of England from seven surveys which provide smoking prevalence estimates and confidence intervals at the LA level, but vary by time, sample size and methodology. Some surveys present synthetic estimates of prevalence at LA level. Synthetic estimation is a model-based technique aimed at combining data obtained from surveys containing the measures of interest with a set of associated covariates at a small area level from another survey which is more powerful in terms of coverage and sample size. For example, Twigg *et al.* (2000) proposed a multilevel modelling approach which estimates the association of demographic characteristics with smoking status, allowing for nesting of individuals within postcode sectors within health authorities. Then synthetic smoking prevalence estimates are obtained by prediction from ecological and individual variables in demographic surveys. Therefore, such prevalence estimates may be based on multiple survey sources (Bajekal *et al.*, 2004; Schenker and Raghunathan, 2007).

The outline of this paper is as follows. Section 2 describes the sources of data that we use and discusses their main features. In Section 3, we present our basic approach to bias adjustment. Section 4 investigates the effect of correlation between sources, Section 5 presents a time trend model and Section 6 describes a classical counterpart to the Bayesian models. Section 7 provides a discussion and conclusion.

## 2. Sources of data

For each of the 48 LAs in the east of England, smoking prevalence estimates and 95% confidence limits are available from four data providers, resulting in seven data sources: the Acxiom data for 2003, 2004 and 2005 (Acx03, Acx04 and Acx05; ), the ‘Action on smoking and health’ (ASH) data for the period 2000–2002 (ASH02; ), the CACI data for 2005 (CACI05; ) and the National Centre for Social Research health profiles (HPs) data for the periods 2000–2002 and 2003–2005 (HP02 and HP05; from the Health Survey for England (HSE), available from the UK Data Archive at the University of Essex, Colchester, and from Office for National Statistics neighbourhood statistics, ).

Acxiom's smoking prevalence estimates are modelled from their National Shoppers Survey, which is a commercial consumer habits postal survey distributed through door drops and by direct mail. The methodology that is used to obtain the estimates is unpublished; possible non-response and coverage problems could be relevant since the final sample is derived from a postal survey. In the CACI data, the estimates are inferred from the commercial ‘Community insights survey’ on household expenditure on tobacco. Detailed information on the survey methodology has not been published, but a simple calculation to derive smoking prevalence from tobacco expenditure has been used (Eastern Region Public Health Observatory, 2007a). This is based on the expenditure on tobacco per person, summed to give expenditure per area, the average retail price of cigarettes, the area population size aged 16 years or older and the average number of cigarettes smoked per smoker per day.

The ASH estimates are provided by ward and are generated by using synthetic estimation techniques based on a multilevel model which incorporates ecological covariates at different levels as suggested in Twigg *et al.* (2004). The underlying source is the HSE. At the first stage, a multilevel model of smoking behaviour using individual terms (age, sex and marital status), area level terms (e.g. percentage of people by social class and percentage of households in rented accommodation) and interaction terms was fitted to the health survey data. At the second stage, the model parameters were applied to the corresponding information that was available for all wards to estimate, in each ward, the proportion of current smokers at each age by sex and marital status group. These estimates were then applied to the corresponding census counts to provide a model-based estimate of current smoking prevalence for all wards in England. The HP estimates are also based on the HSE. A synthetic estimation approach to producing prevalence estimates for each LA in England was used because the sample size of national surveys such as the HSE was too small to provide reliable estimates at a small area level (Scholes *et al.*, 2007). A similar multilevel methodology to that of the ASH estimates was used.

All these estimates are affected by different types of bias, and in several cases details of the methodology that was used to derive them are lacking. The HSE-derived estimates can be considered more reliable since the sampling plan and the methodology that was used for estimation are known. However, HSE results are not immediately available and are published with 1 or 2 years’ delay. Although commercial estimates lack transparency in methodology, they can be more useful when constant monitoring is required (Jones and Tocque, 2005). Moreover, commercial surveys have seemingly larger sample sizes than the HSE at the LA level and therefore potentially greater precision. Our proposed methodology aims to address, in a generic way, any modelling biases that are introduced by the surveys in producing their prevalence estimates.

**Fig. 1:**
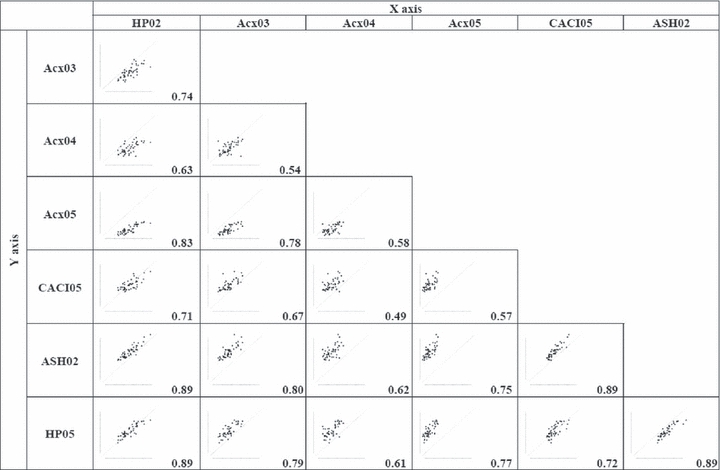
Scatter plots and correlation coefficients of smoking prevalence estimates in 48 LAs between sources of data: the vertical and horizontal axes range from 10% to 35% in every case

**Fig. 2:**
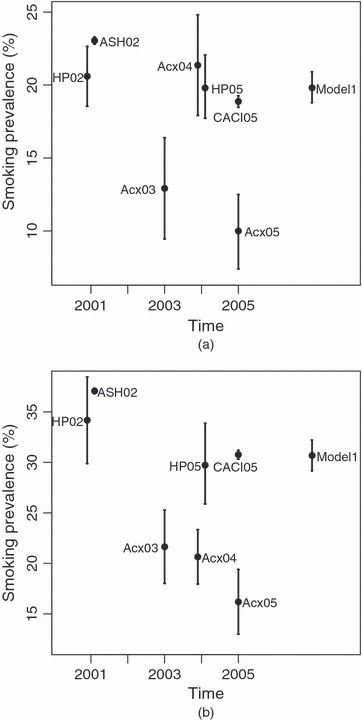
Smoking prevalence estimates and 95% credible intervals from the sources of data, according to (midpoint) date for the survey and model 1: (a) South Norfolk LA; (b) Norwich LA

LA smoking prevalence estimates across the seven data sources are positively correlated (Fig. 1): correlation coefficients range between 0.49 and 0.89. The commercial sources give generally lower estimates than the HSE-based sources. When the estimates from different sources are plotted together, as in Fig. 2 for the case of two particular LAs, the heterogeneity of the estimates and the different precisions are manifest.

## 3. Initial models allowing for bias

The main objective of this work is to develop a general method for obtaining prevalence estimates by using sources of data that are subject to various biases. This section describes a basic Bayesian model and some variants. Let *y*_*ij*_ be the smoking prevalence percentage estimate obtained from data source *j* (*j*=1,…,7) for LA *i*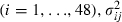 its sampling variance (which is assumed known) and *θ*_*i*_ the ‘true’ underlying smoking prevalence for each LA. *σ*_*ij*_ was obtained by dividing the width of the stated 95% confidence interval by 3.92. Rather than assuming that *y*_*ij*_ is an unbiased estimate of *θ*_*i*_, we introduce an additional additive bias *δ*_*i*_ such that the expectation of

*y*_*ij*_ is *θ*_*i*_+*δ*_*ij*_. Each *δ*_*ij*_ represents the total bias for data source *j* and LA *i*, which may for example include non-response and selection biases as well as model-related biases. We assume a normal sampling model for the *y*_*ij*_, although for small samples more precise binomial models could be adopted. The corresponding biases are assumed ‘exchangeable’ across LAs within data sources, in the sense that we have no reason to think that the bias in any particular LA is systematically different from any other, and hence *δ*_*ij*_ can be considered as random effects which we assume normally distributed. We wish to estimate the bias mean *μ*_*j*_ and the bias standard deviation *τ*_*j*_ for each source of data *j*.

### 3.1. Models

The basic model proposed is 
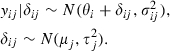
(1)

We shall in general adopt a Bayesian approach with vague priors 
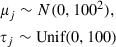
 where *μ*_*j*_ and 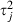 represent respectively the mean bias and the bias variance for data source *j*.

Writing model (1) marginally, we obtain 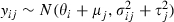
(2) and an identifiability issue becomes obvious because, for example, all *θ*_*i*_ could be increased by an arbitrary constant and all *μ*_*j*_ decreased by the same constant without changing the fit of the model; we note that model (1) resembles a weighted two-way analysis of variance (ANOVA) (Eberly and Carlin, 2000). To implement the model, we therefore need to provide additional information about the *θ*s in addition to equations (1). We introduce an overall smoking prevalence for the east of England and provide this parameter on the basis of external information. It is taken as 23% from the UK General Household Survey for the east of England in 2005 (Goddard, 2006): 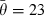, i.e. 
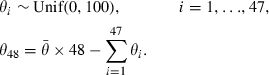


We refer to this assumption, together with equations (1), as *model 1*.

### 3.2. Weighting of sources of data, adjusted estimates and model fit

The main objective of our method is to estimate *θ*_*i*_ for each of the 48 LAs. From distribution (2) we have that 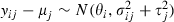 and so, for known *μ*_*j*_ and 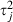, we would be essentially carrying out a fixed effect meta-analysis with 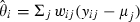, where 
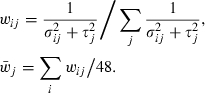
(3)

For LA *i*, *w*_*ij*_ is the weight corresponding to source *j*; 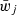 is the average of *w*_*ij*_ across LAs for source *j*. We can therefore assess how much each source is weighted in the pooled analysis by monitoring standard random-effects meta-analysis weights for each source of data and taking averages across LAs. This is useful in reflecting the relative precisions of the bias-adjusted estimates and to understand the overall results being obtained.

For each LA *i* and source of data *j*, we can also consider a ‘bias-adjusted estimate’
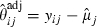
(4) with variance 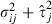, where 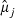 and 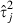 are estimates, which here are taken as posterior means. The 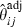
s are useful to show how our technique works, in the sense that they reveal the half-way stage of the procedure towards the pooled results.

To assess model fit, we calculate the standardized residuals 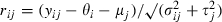 and their sum of squares 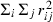. We also compare models by using the deviance information criterion DIC as a measure of predictive ability, in which model fit is penalized by model complexity (Spiegelhalter *et al.*, 2002). Compared with one model, an alternative model with a DIC lower by more than about 2 is judged more appropriate for the data.

### 3.3. Results

To implement the model we used WinBUGS software (Spiegelhalter *et al.*, 2007) which generates Markov chain Monte Carlo simulations of the resulting posterior distributions. The number of iterations for the burn-in period and the overall number of iterations ranged from 10000 to 90000 and from 20000 to 110000 respectively. The number of the chains used ranged from 1 to 3. Convergence was checked by using several initial values for each of the chains. However, since convergence performance was not always satisfactory, we used *redundant parameters* (Gelman and Hill (2007), pages 420–421) in the form 
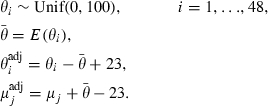


We monitored 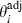 and 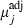 instead of *θ*_*i*_ and *μ*_*j*_, obtaining convergence much faster. Results are presented as posterior means and 95% credible intervals (CIs). Monte Carlo errors ranged up to 3% of posterior standard deviations.

Fig. 2 shows the smoking prevalence estimates for two LAs (with low and high prevalence). The posterior mean and 95% CI from model 1 above are shown together with the smoking prevalence estimates and 95% confidence intervals from the original data sources. The latter are plotted according to the (midpoint) date for the survey; we consider a model for trend over time in Section 5. For these two LAs, and typically of all LAs, the pooled prevalence estimates from model 1 are close to those from the HP02, HP05 and CACI sources of data. There were no extreme outliers among the standardized residuals, and the sum of squared standardized residuals (posterior mean 313; standard deviation (SD) 22) was compatible with a null *χ*^2^-distribution on 48×7=336 degrees of freedom.

The HP02, HP05 and ASH02 estimates are all based on the HSE. So these estimates are expected to have similar variability. In fact, the ASH02 estimates seem potentially overprecise, and we also suspect overprecision in the CACI05 estimates. To assess whether this has an effect on the estimates from model 1 we enlarged the original confidence intervals for the ASH02 and CACI05 estimates, by setting their standard errors (SEs) equal to the SE for the corresponding original HP02 estimate and then rerunning model 1 on the new data. There were no systematic changes in the parameters which represent mean bias, or the prevalence estimates for individual LAs. Lower posterior mean estimates were obtained for all bias variances, these being substantially lower for the ASH02 and CACI05 estimates. So, although the reported confidence intervals are not entirely believable, an advantage of our methodology is that, by incorporating the bias variance 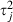, we largely compensate for any systematic underestimation or overestimation of the SEs. The methodology for calculating the 95% confidence intervals would ideally be available; in the absence of such details the reported confidence intervals should be treated with caution and the effects of underestimation considered. As this example shows, however, the methodology appears to be able to cope with some misestimation of the SEs.

**Fig. 3:**
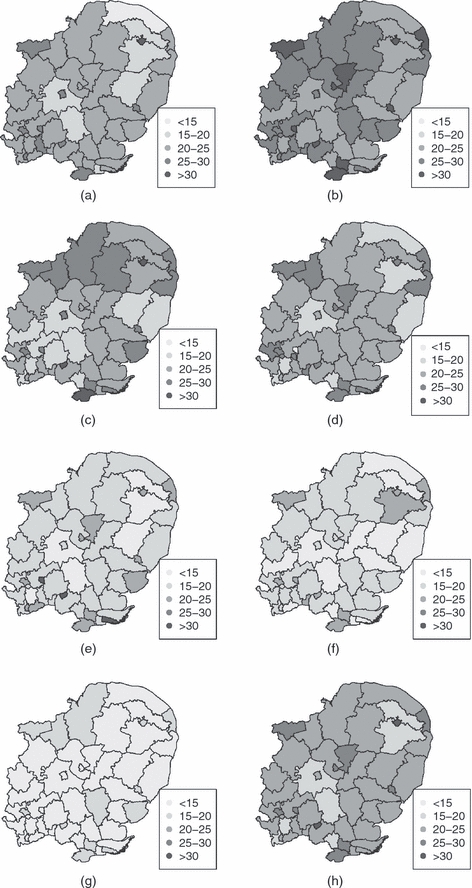
Smoking prevalence estimates for the 48 LAs in the east of England from the original sources of data and model 1, tinted according to five ranges: (a) CACI05; (b) ASH02; (c) HP02; (d) HP05; (e) Acx03; (f) Acx04; (g) Acx05; (h) model 1

**Fig. 4:**
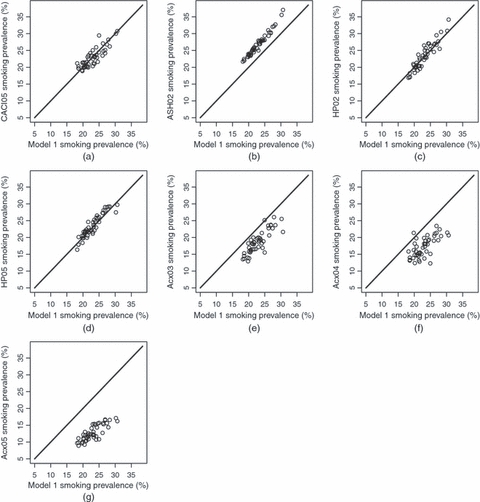
Model 1 smoking prevalence estimates plotted against the original source of data estimates, for all 48 LAs: (a) model 1 *versus* CACI05; (b) model 1 *versus* ASH02; (c) model 1 *versus* HP02; (d) model 1 *versus* HP05; (e) model 1 *versus* Acx03; (f) model 1 *versus* Acx04; (g) model 1 *versus* Acx05

Fig. 3 outlines a geographical comparison of LAs in the east of England for the original data source estimates and model 1 estimates, shaded according to five smoking prevalence ranges. The posterior for each *θ*_*i*_ reflects contributions from all the sources of data. Fig. 4 shows the smoking prevalence estimates of model 1 plotted against the original data source estimates. The CACI and the HP estimates are closer to the model 1 estimates, indicating that they incorporate a smaller amount of bias. The ASH source tends to overestimate smoking prevalence, whereas the Acxiom sources tend to underestimate.

**Fig. 5:**
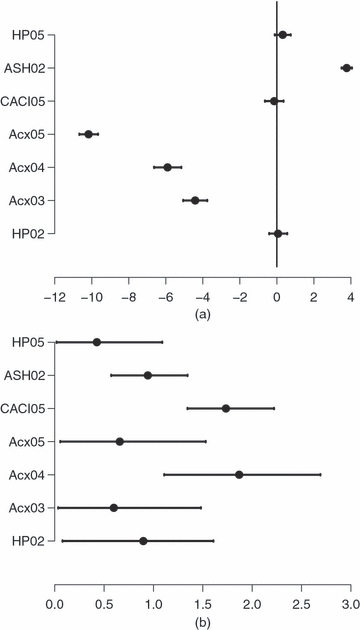
(a) Model 1 smoking prevalence mean bias μ_*j*_ and (b) SD of bias τ_*j*_ (posterior means and 95% CIs) for the various sources of data

**Fig. 6:**
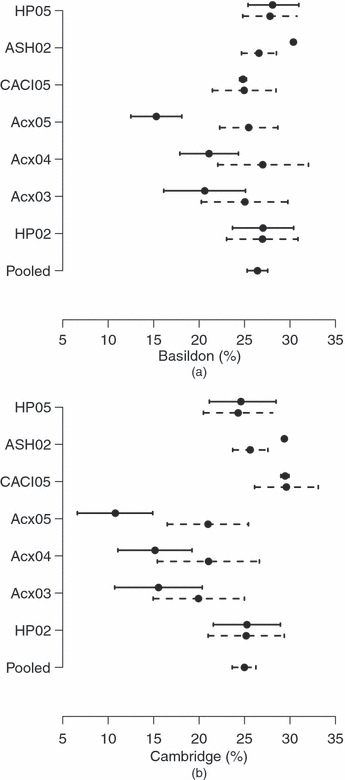
Smoking prevalence estimates with 95% CIs for (a) Basildon and (b) Cambridge LAs: 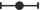, original estimates; 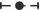, adjusted source-specific estimates; 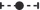, model 1 posterior pooled estimates

This is confirmed by the plots in Fig. 5, which presents a direct comparison of the posterior mean and standard deviation of the bias for each source of data obtained from model 1, together with their precisions. In Fig. 6 adjusted source-specific estimates are plotted alongside the original estimates, together with the pooled smoking prevalence estimates, for Fenland and Cambridge LAs (using model 1). The original estimates are shifted towards the pooled estimate by the estimated bias for each source of data, and the CI is widened, reflecting the bias variance for that source.

Posterior distributions for the average weights in expression (3) are shown in Table 1. The ASH02 distribution has the highest average weight (0.39), followed by HP05 (0.16) and by HP02 (0.12). In total, the commercial surveys (Acxiom and CACI) receive a total weight averaging 0.33.

**Table 1:** Results for average weights in models 1 and 2 (posterior mean and 95% CIs)

Source of data	Weights (CIs) averaged across LAs	
	
	Model 1	Model 2
ASH02	0.39 (0.22,0.62)	0.34 (0.19,0.57)
Acx03	0.06 (0.04,0.08)	0.07 (0.04,0.09)
Acx04	0.05 (0.03,0.09)	0.06 (0.03,0.10)
Acx05	0.11 (0.05,0.15)	0.10 (0.05,0.15)
CACI05	0.11 (0.06,0.17)	0.08 (0.05,0.13)
HP02	0.12 (0.06,0.19)	0.17 (0.08,0.23)
HP05	0.16 (0.09,0.21)	0.19 (0.12,0.24)

The overall regional prevalence figure of 23% itself has some imprecision, having a reported SE of 1.1% (Eastern Region Public Health Observatory, 2007b). We can incorporate this uncertainty by writing 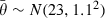. However, this again reintroduces the problems of identifiability of the *θ*_*i*_, since the average of the *θ*_*i*_ is no longer firmly anchored, and makes convergence problematic. Although the uncertainty in each individual *θ*_*i*_ is increased, the differences between LA prevalence estimates, *θ*_*i*1_−*θ*_*i*2_, and their uncertainties are unaffected. Similarly the differences between LA prevalence estimates are unchanged if the overall prevalence is changed from 23% to another fixed number.

### 3.4. Alternative models

Some alternative models for the data were explored, as follows. Firstly, knowledge about the reliability of sources of data can add useful information. So, instead of using an overall regional prevalence, we could assume that one of the sources of data (e.g. HP02) is believed to be ‘unbiased’. This could have various definitions in terms of *μ*_*j*_ and 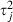, but here we consider assumptions on *μ*_*j*_ only so that the source of data is on average unbiased but biases may exist in individual LAs. Together with equations (1), we write *μ*_HP02_=0; *θ*_*i*_∼Unif(0,100), *i*=1,…,48, where *μ*_HP02_ is the mean bias for the HP02 source. The LA prevalence estimates from this model were very similar to that from model 1, as might be expected given the estimated mean bias of near 0 for HP02 in Fig. 5. The adequacy of this alternative model which fixes the mean bias for HP02 to be 0 (DIC 1014.7) appears to be similar to that of the original model 1 (DIC 1015.8).

As a further level of sophistication, we consider a hypothesis of exchangeability (i.e. similarity) for the bias variance parameters to obtain potentially narrower CIs and shrinkage of the variance estimates through a degree of pooling information (Spiegelhalter *et al.*, 2004). We assume a half-normal distribution for *τ*_*j*_ (the remaining assumptions are those of model 1) by writing 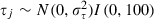 and *σ*_*τ*_∼Unif(0,100) where *I*(*c*_1_,*c*_2_) is the indicator function taking the value 1 in the interval (*c*_1_,*c*_2_) and 0 otherwise. In the final model, we assume equality of the bias variance parameters 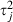, writing *δ*_*ij*_∼*N*(*μ*_*j*_,*τ*^2^), *τ*∼Unif(0,100). Again these two models produced very similar LA prevalence estimates to those of model 1. However, they are not appropriate for our data since their DICs of 1018.6 and 1029.9 respectively are larger than that for model 1. However, these models could be useful in examples with fewer areas.

## 4. Addressing correlation between sources

As discussed in Section 2, some of the estimation from different sources of data, namely HP and ASH, was carried out using similar methodology and on the basis of the same underlying survey, whereas for other estimates (the CACI05 and the Acxiom estimates) little information about the technique used was available. Any correlation between sources could affect our bias-adjusted estimates. For example, if the estimated correlation coefficient between two sources of data is close to 1, then they essentially contribute only one piece of information. In this section, we discuss the implementation of a model which estimates the correlation coefficients between pairs of sources of data.

### 4.1. Model including correlation between sources of data

We address the correlation between sources of data by making the *δ*_*ij*_ multivariate. For example, for two sources *j* and *k*, we use a bivariate normal distribution: 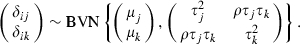 To model the 7×7 correlation matrix for seven sources of data we use the scaled inverse Wishart model (O'Malley and Zaslavsky, 2005) and extra multiplicative parameter factors to give faster convergence (see Gelman and Hill (2007), pages 286–287 and pages 376–377 for details). As in model 1, we assess how much each source is weighted in the pooled analysis, monitoring standard random-effects weights for each source of data and taking an average across LAs. We also obtain bias-adjusted estimates and CIs, as in expression (4). The resulting model with the usual constraint 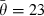 is therefore as follows (assumptions and notation which are in common with model 1 have been omitted): 
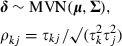
 where *ρ*_*kj*_ is the correlation coefficient between sources *k* and *j*, and *τ*_*kj*_ is the generic element of matrix Σ (with 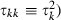. We refer to this as *model 2*.

### 4.2. Results

Table 2 shows the estimated correlation matrix. The CACI05 and ASH02 sources of data are found to be positively correlated, with the corresponding 95% CI excluding zero. Although some of the remaining correlations between sources of data have moderately high estimates, the corresponding 95% CIs are wide and all include zero.

**Table 2:** Between-sources correlation coefficients from model 2 (posterior means and 95% CIs)

Source of data	Correlation coefficients for the following sources of data:						
	
	ASH02	Acx03	Acx04	Acx05	CACI05	HP02	HP05
ASH02	1						
Acx03	0.03 (−0.78,0.78)	1					
Acx04	−0.20 (−0.72,0.38)	0.22 (−0.61,0.84)	1				
Acx05	0.20 (−0.79,0.48)	0.35 (−0.58,0.90)	0.53 (−0.07,0.89)	1			
CACI05	0.61 (0.21,0.85)	0.23 (−0.71,0.89)	0.18 (−0.34,0.61)	0.23 (−0.48,0.76)	1		
HP02	−0.10 (−0.82,0.78)	−0.08 (−0.87,0.83)	−0.08 (−0.80,0.77)	−0.10 (−0.84,0.79)	−0.30 (−0.93,0.82)	1	
HP05	0.09 (−0.79,0.86)	0.05 (−0.82,0.87)	−0.03 (−0.80,0.79)	−0.02 (−0.83,0.84)	0.00 (−0.88,0.89)	0.08 (−0.83,0.90)	1

Table 1 reports the weights that were assigned to each source in model 2. The ASH02 source has the highest average weight (0.34), followed by HP05 (0.19) and by HP02 (0.17). Comparing the weights estimates in models 1 and 2, the ordering and magnitude of the weights among data sources is similar. The weights for the ASH02 and CACI05 sources are slightly lower in model 2 than in model 1, whereas weights for HP02 and HP05 are higher. This makes sense in light of the fitted correlations in model 2. The highest correlation is that between ASH02 and CACI05, so these sources are expected to receive less weight individually once correlations have been acknowledged. HP02 and HP05 showed the lowest correlations with other sources of data, so their relative weight in the analysis increases.

Including the correlations hardly affects the LA prevalence estimates: for example, the model 1 estimate for Fenland is 24.59 (95% CI 23.42–25.80), whereas the model 2 estimate is 25.31 (95% CI 24.04–26.69). The CIs for the prevalence estimates are slightly wider (the posterior SDs are increased proportionally by an average of 4.8% in model 2 compared with model 1), reflecting the acknowledgement that the sources of data are not independent pieces of information. Model 2 is a better representation of the data than model 1, with DIC 1001.0 compared with 1015.8.

## 5. Time trend model

Our aim so far was to find a general approach for combining information from various sources of data. The information was modelled hierarchically, and the time period to which the source

estimates related was ignored. However, this additional information could be used in developing a linear time trend model aimed at obtaining forecasts for future levels of smoking prevalence in each LA. In our example of smoking prevalence survey data with its limited time span, such modelling might not be very informative. We nonetheless pursue this analysis, since in other examples it could be more practically useful.

For each LA *i*, we assume a linear trend with time with intercept *α*_*i*_ and slope *β*_*i*_; the *α*_*i*_ are considered as fixed effects and the *β*_*i*_ as random effects. The random slopes allow for LA-specific linear divergence from the overall regional trend.

### 5.1. Model specifications

To fit this model, the sources of data need to be labelled with the times when the corresponding surveys were conducted. A mid-survey time point was chosen for data sources HP02, HP05 and ASH02. Let *x*_*ij*_ be 2005 minus the year of source *j*. On the basis of model 1, but with the assumption 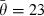 concerning the regional constraint for 2005 now transferred to the average of the *α*_*i*_, we propose the following model: 
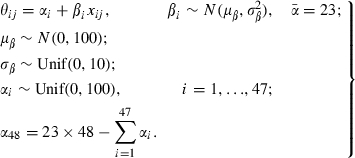
(5)

We refer to this as *model 3*.

### 5.2. Results

**Fig. 7:**
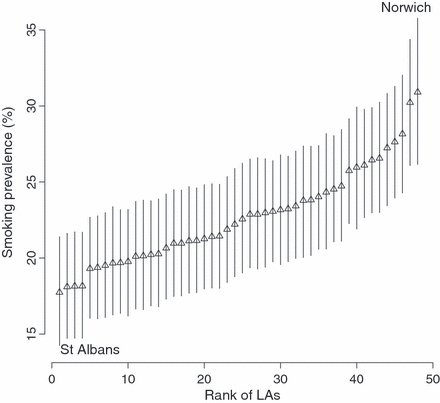
League table of LAs according to 2009 smoking prevalence predictions (with 95% CIs) from model 3

Model 3 gave posterior means (with SDs in parentheses) for *μ*_*β*_ and *σ*_*β*_ of −0.08 (0.25) and 0.14 (0.09) respectively. So, on average, the percentage smoking prevalence is estimated to decrease by 0.08 per year, and the SD of individual LA slopes around this average is 0.14 per year. Fig. 7 is a league table plot of estimates and CIs from model 3 for LAs in the east of England in 2009. Norwich is expected to be the LA with the highest smoking prevalence, whereas St Albans is predicted to be the LA with the lowest smoking prevalence; the CIs represent the uncertainties in the predicted LA smoking prevalences, and thus in their expected ranking. These forecasts of course also assume continuation of the linear trends that were observed in recent years.

## 6. Classical approach

Here we present a classical analysis of the data that were described in Section 2 as a counterpart to the Bayesian analyses of the previous sections. In particular, we want to obtain a classical model to be compared with the basic model 1. We implement this first by using a simple iterative ANOVA approach (Section 6.1), and then by using the mixed effects modelling tools that are provided by the R and the S-PLUS packages (Section 6.2). Attempts to set up the correct variance structure for model 2 in a classical framework were not successful.

### 6.1. Iterative analysis-of-variance algorithm

We propose an iterative procedure based on alternating a simple ANOVA model with performing a random-effects meta-analysis which provides a moment estimator of 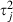. This gives a computational method which reaches convergence very quickly. The procedure is carried out with the following steps.

*Step 1* (ANOVA step): perform a weighted two-way ANOVA of prevalences by LA and survey to give estimates of *θ*_*i*_ and *μ*_*j*_. Weights are 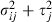. Initially 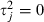.
*Step 2* (external information step): rescale the average *θ*_*i*_ to 23%.
*Step 3* (meta-analysis step): calculate the values 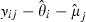 with assigned variance 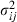. For each survey *j*, perform a random-effects meta-analysis providing a moment estimator of 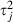.
*Step 4* (iteration step): go to step 1 and iterate until convergence.
*Step 5* (mean-square error adjustment step): adjust the final SEs by a multiplicative factor (Thompson and Sharp, 1999) because the mean-square error is equal to 1 when the weights are the reciprocals of variances as in this case.

This algorithm was implemented in the R package (R Development Core Team, 2009).

### 6.2. Mixed effects model

Model 1 is a mixed effects model in which the biases *δ*_*ij*_ are random effects and the prevalences *θ*_*i*_ are fixed effects, which can be written as 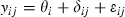
(6) where 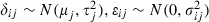 and the variances 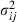 are assumed known. Alternatively we can rewrite equation (6) as 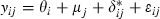
(7) where *θ*_*i*_+*μ*_*j*_ is the fixed effects part and 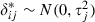 is the random-effects part of the model. We refer to this as *model 4*. The new formulation (7) is equivalent to equation (2) and is a mixed effects model with the peculiarity of having a fixed and known variance structure for the within-group errors, derived from the confidence intervals that are provided by each survey for each LA. The implementation details and relevant S-PLUS code are presented in Appendix A.

### 6.3. Results and comparison with Bayesian analyses

**Fig. 8:**
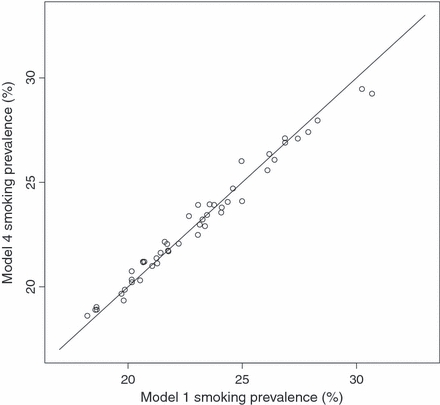
Model 4 *versus* model 1 smoking prevalence estimates for 48 LAs

Convergence from the iterative algorithm that was presented in Section 6.1 is reached within 10 iterations only, and the final estimates from this and from model 4 are very close to those obtained with model 1. In Fig. 8, model 4 smoking prevalence estimates are plotted against model 1 smoking prevalence estimates.

The advantages of the Bayesian approach include the ability to implement model 2. This model is preferred both in principle because it allows for potential correlations between sources of data and by virtue of its better fit to the data compared with model 1. Such a model was not successfully fitted by using a classical analysis. Other advantages of Bayesian modelling include the ease of obtaining predictions and associated uncertainty intervals (as in Section 5), allowance for the uncertainty in 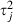 (which is not acknowledged in the above classical analysis), having a convenient measure for model comparison (namely DIC), and the possible extension to incorporate relevant prior information or beliefs. However, it does have some disadvantages, in that it requires specialist software and the difficulties that are sometimes encountered in achieving Markov chain Monte Carlo convergence.

## 7. Discussion

In this paper, we have proposed a general method to combine prevalence estimates from multiple sources of data. Bayesian approaches to regional prevalence estimation have become popular in recent years (Branscum *et al.*, 2008). Our method is based on finding suitable additive Bayesian hierarchical models to address the various biases, so that both combined prevalence estimates and the amount and variability of bias in each source of data can be quantified. We used smoking prevalence estimates that are available at the LA level. Estimates from different sources, obtained with different methodologies, were combined and an analysis of possible correlation between sources of data was carried out. Finally, a time trend model was implemented. These methods could be extended, for example, to incorporate informative priors for the bias variances 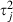 to capture beliefs or empirical evidence about the reliability of the various sources of data. Alternative models that assume proportional rather than additive biases could have been developed by similar modelling of log(*y*_*ij*_) rather than *y*_*ij*_ itself.

Published classical approaches to combining information from multiple surveys are the multiple-frame method (Hartley, 1974; Lohr and Rao, 2000, 2006) and the statistical matching method (Kadane, 2001; Rodgers, 1984; Moriarity and Scheuren, 2001), whereas a scoring method was recently proposed by Elliott and Davis (2005). The basic idea in multiple-frame estimation is that different sampling frames (possibly overlapping), whose union covers the whole population, are considered and probability samples are drawn independently from each frame. Samples are then properly combined to obtain optimal linear estimators of population quantities. Statistical matching considers records of subjects having ‘similar profiles’ from different sources of data and puts together the information from them. For example (see Rodgers (1984)), one of two data sets could consist of information about healthcare expenses whereas the other data set could consist of information about welfare benefits received. By matching similar individuals, one can analyse variables from the first data set together with variables from the second data set to explore the association between healthcare expenses and welfare benefits. Elliott and Davis (2005) proposed a method which is based on adjusting the weights from two surveys such that the complementary strengths of each survey in terms of sample size or unbiasedness are exchanged. One survey may be less biased but does not have small area identifiers, whereas the other survey may have larger sample sizes but suffer from more bias.

Bayesian small area prevalence estimation for combining multiple sources has been recently considered by others. Elliott and Little (2000) proposed some general principles for model selection in a Bayesian framework. Jackson *et al.* (2008) presented Bayesian graphical models for fitting a common regression model to multiple data sets with different levels of aggregation. These models were applied to a study of low birth weight and air pollution in England and Wales by using register, survey and small area aggregate data. In recent work by Raghunathan *et al.* (2007) small area estimation was addressed by combining prevalence rates from two surveys, which differ in bias, coverage and sample size, by means of a hierarchical Bayesian approach.

Our approach is similar but more general and, above all, it is based on combining aggregate prevalence rates rather than modelling individual level data. Our methods provide estimates of smoking prevalence in each area, based essentially on meta-analysis of synthetic estimates whose methodology is often not sufficiently described. It would be preferable to have more details of the sampling, estimation and variance calculation, or in addition actual prevalence data obtained from a direct survey. In the latter case, our results could be used to provide prior distributions for each LA, which would help in reducing the sample size that is required and the costs of the survey. The sources of data that we considered are not independent, so it is reasonable to include the correlations. For these reasons, model 2 is our preferred model, but we recognize that convergence can be problematic.

Knowledge of smoking prevalence at a small area level is essential for the development of effective health promotion strategies. Bias modelling helps us to acknowledge and adjust for the discrepancies in location and precision between estimates that are provided by different sources. The flexibility of our approach allows its application to other areas where prevalence rates estimation is a key target for policy decision makers. Topical examples that are related to healthcare and social affairs include obesity and physical activity, human immunodeficiency virus infection, illegal drug use and crime rates.

## Appendix A: Implementation of model 4

We tried to implement model 4 in R (R Development Core Team, 2009), since R can handle variance functions for modelling this type of heteroscedasticity by using the varFixed class in the lme() function of package nlme or the weights option in the lmer() function of package lme4. However, when the within-group variance is assumed known from the data set, as is in our case, these options are not useful since they model the variance when the within-group-variance is known *up to a constant of proportionality* (Pinheiro and Bates (2000), page 208). Following Ng (2005), pages 12–13 and 23, we can model the known variances by using the sigma=1 setting for the lmeControl option. This is implemented for the lme() function in S-PLUS only, not in R. Furthermore, to achieve convergence we use the so-called *treatment contrasts* as in R, instead of the *Helmert contrasts* which are the default in S-PLUS.

The S-PLUS code for the analysis in Section 6.2 is as follows:

Table<- read.table(paste(path,“/SPDataMixedEffectsModel.csv”, sep =“”), sep=“,”,header=TRUE, as.is=TRUE)

Table$AUTHORITY<-as.factor(Table$AUTHORITY)

Table$SURVEY<-as.factor(Table$SURVEY)

TABLE<-data.frame(Table)

options(contrasts = c(factor =“contr.treatment”, ordered =“contr.poly”))

MixEffect.lmeFinal<-lme(PREVALENCE∼(AUTHORITY + SURVEY), data = TABLE, random =∼SURVEY|AUTHORITY, control = lmeControl(sigma = 1), weights = varFixed(∼VAR))

in which path is the directory path where the data set SPDataMixedEffectsModel.csv is stored.

In the file SPDataMixedEffectsModel.csv, data are organized according to the two factors, AUTHORITY and SURVEY. The random-effects component is (AUTHORITY + SURVEY), whereas a random SURVEY effect is grouped by AUTHORITY.
